# Polymerase chain reaction–based assays for the diagnosis of human brucellosis

**DOI:** 10.1186/s12941-014-0031-7

**Published:** 2014-08-01

**Authors:** Ying Wang, Zhanli Wang, Yaxian Zhang, Liyun Bai, Yue Zhao, Chunfang Liu, An Ma, Hui Yu

**Affiliations:** 1 Baotou Medical College, Baotou 014010, China; 2 Zhejiang Academy of Medical Sciences, Hangzhou 310013, China

**Keywords:** Polymerase chain reaction techniques, Human brucellosis, Brucella spp, Molecular diagnosis

## Abstract

Polymerase chain reaction (PCR) is an in vitro technique for the nucleic acid amplification, which is commonly used to diagnose infectious diseases. The use of PCR for pathogens detection, genotyping and quantification has some advantages, such as high sensitivity, high specificity, reproducibility and technical ease. Brucellosis is a common zoonosis caused by Brucella spp., which still remains as a major health problem in many developing countries around the world. The direct culture and immunohistochemistry can be used for detecting infection with Brucella spp. However, PCR has the potential to address limitations of these methods. PCR are now one of the most useful assays for the diagnosis in human brucellosis. The aim of this review was to summarize the main PCR techniques and their applications for diagnosis and follow-up of patients with brucellosis. Moreover, advantages or limitation of the different PCR methods as well as the evaluation of PCR results for treatment and follow-up of human brucellosis were also discussed.

## Introduction

Brucellosis is a widespread zoonotic disease caused by members of genus *Brucella*. Its prevalence is more than 10 per 100 000 population in some endemic countries [1]. Transmission of brucellosis from animals to humans occurs mainly through direct contact with infected animals, ingestion of raw dairy products of animal origin, or consumption of infected meat from domestic livestock [2]. Human brucellosis may lead to a variety of clinical presentations, such as fever, sweating, chills, headache, malaise, myalgia and even arthralgia of the large joints [3]. The presentations and phases of the disease may be acute, sub-acute, chronic, relapsed, active or inactive. Antibiotic treatment of human brucellosis often results in high treatment failure and relapse rates. Because the clinical presentation is non-specific, laboratory testing is required for confirmation.

*Brucella* species are gram-negative, facultative intracellular bacteria, which lack capsules, flagellae, endospores or native plasmids [4]. Currently, the genus *Brucella* consists of ten species: *B. abortus, B. suis, B. ovis, B. melitensis, B. canis, B. neotomae, B. pinnipedialis, B. ceti, B. microti* and *B. inopinata*[5]. Four species of the genus *Brucella* are pathogenic for humans, namely *B. melitensis*, *B. abortus*, *B. suis*, and *B. canis. B. melitensis* is considered as the most pathogenic species, followed by *B. suis*, whereas *B. abortus* is the mildest type of brucellosis. Additionally, *Brucella* isolates from marine mammals can cause human infections. DNA-DNA hybridization studies demonstrated that there are high degrees of genetic similarity of *Brucella* spp. [6].

At present, there are various assays for diagnosis of human brucellosis such as standard microbiological tests for the isolation of *Brucella* spp. from blood, tissue specimens, body fluids and bone marrow, serological tests for the detection of anti-*Brucella* spp. antibodies and molecular methods for the detection of *Brucella* spp. DNA [7]. The most commonly used methods for detection and segregation of *Brucella* spp. were culture techniques and serological tests (standard agglutination tube test, anti-human globulin test, Rose-Bengal test, mercaptan-based tests, enzyme-linked immunosorbent assay and brucellacapt) [8],[9]. However, isolation of *Brucella* spp. is associated with a risk of laboratory-acquired infections and time consuming, and culture sampling sensitivity is often low, depending on the culture medium, *Brucella* species, disease stage and quantity of circulating bacteria. Serological tests seem to be more effective but can be unspecific due to cross reaction or subsensitive reactions in samples from areas with a low or subclinical prevalence of brucellosis [10]. The principles and the main applications of these methods have been well reviewed elsewhere [11].

To ensure effective brucellosis disease prevention and control, a fast and accurate identification method is necessary. Polymerase chain reaction (PCR) technique offers a sensitive and specific way of detecting *Brucella* spp. from peripheral blood and other tissues [12]. The first brucellosis PCR-based test was introduced by Fekete *et al*. in 1990 [13]. They successfully amplified a 635 bp fragment of a 43 kDa outer membrane protein gene from *B. abortus* strain 19. Several studies have reported that PCR is a very useful tool for the rapid diagnosis of acute brucellosis and a good marker for the posttreatment follow-up and the early detection of relapses [14],[15]. Moreover, many studies have developed PCR-based assays to differentiate 10 *Brucella* species [16]–[18]. Up to data, PCR assays have been used in diagnosis of both animal brucellosis and human brucellosis [19]. To our current knowledge, at least 200 reports have been published dealing with various methods based on PCR for laboratory diagnosis of human brucellosis. This review article highlights various PCR-based methods for the clinical diagnosis of human brucellosis. The principles, advantages or limitation of the different methods are also being discussed together with examples of applications taken from the literatures.

### Standard PCR

For the diagnosis of human brucellosis, a PCR assay with one pair of primers was developed, which amplifies the target genomic sequence of *Brucella* species. Primer pairs include the primers for sequences encoding 16S rRNA [20],[21], outer membrane protein (omp2a, omp2b) [22]–[24], 31-kDa immunogenic *Brucella abortus* protein (BCSP 31) [25],[26], 16S-23S ribosomal DNA interspace region [27] and insertion sequence (IS711) [28],[29]. Studies showed that standard PCR appeared to be a more sensitive technique than microbiological methods, not only for the diagnosis of a first episode of infection, but also for the early detection of relapses [30]–[32]. Some research groups also assessed the performances of standard PCR as diagnostic tools for human brucellosis with respect to conventional methods. Their results showed that standard PCR is a promising diagnostic tool for patients with clinical signs and symptoms, and negative serological results, allowing an accurate and early diagnosis of human brucellosis [33],[34].

The standard PCR is simple and efficient. However, efficiency of this method is dependent on the specificity of the primers. Different primer pairs have previously been published for *Brucella* spp. detection, and only a few of them have been used in human samples. Baddour MM *et al*. compared sensitivity of 3 pairs of primers amplify 3 different fragments including a gene encoding BCSP 31 (B4/B5), a sequence 16S rRNA of *B. abortus* (F4/R2), and a gene encoding omp2 (JPF/JPR). The results showed that the sensitivity of the B4/B5 primer pair, JPF/JPR primer pair and F4/R2 primer pair was 98%, 88.4% and 53.1%, respectively [35]. Navarro *et al.* also compared PCR methods using these 3 pairs of primers as described above. Their results further indicated that the three primers assayed showed a difference in sensitivity by the presence of human genomic DNA [36]. Table 1 showed the efficiency of blood PCR assays using different primers.

**Table 1:** Efficiency of blood PCR assays

**Primer**	**Sequence**	**Target gene**	**Sensitivity**	**Specificity**	**Positive predictive value**	**Negative predictive value**	**Reference**
B4	TGGCTCGGTTGCCAATATCAA	bcsp31	100	100	100	98	14
B5	CGCGCTTGCCTTTCAGGTCTG						
JPF	GCGCTCAGGCTGCCGACGCAA	omp2	98	100	100	96.1	14
JPR	ACCAGCCATTGCGGTCGGTA						
P1	TGGAGGTCAGAAATGAAC	omp2	99	100	100	98	14
P2	GAGTGCGAAACGAGCGC						
26A	GCCCCTGACATAACCCGCTT	bp26	98.5	100	100	97.1	14
26B	GAGCGTGACATTTGCCGATA						
F4	TCGAGCGCCCGCAAGGGG	16S	53.1	100	53.1	100	35
R2	AACCATAGTGTCTCCACTAA	rRNA					

In fact, blood samples are often used for the diagnosis of human brucellosis by the standard PCR [37]. Several factors were reported to affect PCR results in a blood specimen such as the high concentrations of leukocytes DNA and heme compounds [38]. Additionally, human genomic DNA affect the sensitivity of peripheral-blood PCR assay for the detection of *Brucella* DNA [36]. Zerva L *et al*. reported that serum samples should be used preferentially over whole blood for diagnosis of human brucellosis by PCR [39], but Mitka S *et al*. revealed that buffy coat and whole blood were the optimal specimens [14]. Moreover, sample volume used and efficient DNA extraction protocol are also the points of concern for the standard PCR to be used in routine laboratory testing for human brucellosis [34].

### Real time PCR

Compared with the standard PCR, real-time PCR is a valuable technique in determining the quantification of nucleic acids in individual blood samples, as well as in automating the data. With the decreasing prices of real-time PCR thermocyclers and the reagents, many more people now have access to this technology to measure DNA copy number, mRNA expression levels and viral titers [40]. Recently, real-time PCR for the rapid detection and differentiation of *Brucella* species in clinical samples has recently been developed, targeting 16S-23S internal transcribed spacer region (ITS) and the genes coding omp25 and omp31 [41], BCSP 31 [42]–[44], and IS711 [45],[46].

Real-time PCR seems to be highly reproducible, rapid, sensitive and specific. Additionally, this assay is easily standardized and minimises the risk of infection in laboratory workers. It is therefore a useful method for both the initial diagnosis of human brucellosis and the differentiation among inactive, seropositive, and active states. Queipo-Ortuño *et al*. reported that the sensitivities of a SYBR Green I LightCycler-based real-time PCR assay with serum samples was 93.3%, which is higher than 90% and 65% obtained by PCR-ELISA with whole blood samples and blood cultures, respectively [47]. This group further developed a LightCycler-based real-time PCR assay to detect *Brucella* DNA in serum samples. This assay was found to be 91.9% sensitive and 95.4% specific when tested with 65 negative control samples and 62 serum samples from patients with active brucellosis [48].

Furthermore, Surucuoglu S *et al*. compared the TaqMan real time PCR technique to conventional methods using serum samples from patients with different clinical forms of brucellosis. The sensitivity, specificity, positive and negative predictive values of this PCR method were calculated as 88%, 100%, 100%, and 83%, respectively [49]. Alsayed Y *et al*. further investigated the potential of a combination of several tests (culture, ELISA and real-time PCR) to support the diagnosis in different clinical manifestations of brucellosis with peripheral blood samples. They found that if the agglutination test is negative, real-time PCR, and/or ELISA, and/or culture are recommended [50]. Moreover, using a panel of seven primer sets, Winchell JM *et al*. developed a real-time PCR method to differentiate members of the *Brucella* genus isolates, and has the potential to detect novel species [51]. Other studies also reported that real-time PCR allowed the rapid diagnosis of human brucellosis [52],[53]. These results suggest that the high species specificity and selectivity of real-time PCR assay make it a useful tool for diagnosis of human brucellosis.

Just as standard PCR, efficiency of real-time PCR is also dependent on the specificity of the primers. Kattar MM *et al*. developed three real-time PCRs for diagnosis of human brucellosis at genus level with hybridization probes and primers from *16S-23S ITS*, *omp25* and *omp31*. Their results showed that real-time PCR with *16S-23S ITS* primers and its probes was the most sensitive, indicating its potential for the diagnosis of human brucellosis in the clinical laboratory [41]. Additionally, a study analyzed the sensitivity and specificity of the 3 established real-time PCR methods using primers and TaqMan probes targeting the *IS711*, *bcsp31* and *per* genes, and it also compared their efficiencies for the detection of the *Brucella* genus. The results showed that the *IS711*-based real-time PCR was the most sensitive, specific and efficient to detect *Brucella* spp. [54]. Table 2 showed that the sensitivity of the *IS711* target was identical or 10 times higher that the sensitivity of the two other targets [54]. Moreover, the influences of other factors involved in the efficiency of the amplification process of real-time PCR for the diagnosis of human brucellosis were also reported, such as immunoglobulin G, which were extracted with the template DNA from serum samples [55].

**Table 2:** Comparison of conventional and real-time PCR assays lower limit of detection (fg)

**Brucella**	**IS711 copy number**	**Conventional PCR**			**Real-time PCR**		
		IS711	bcsp31	per	IS711	bcsp31	per
B. canis RM6/66	6	100	1000	1000	2	20	20
B. abortus 544	7	100	1000	1000	2	2	2
B. melitensis 16 M	7	1000	1000	1000	2	20	20
B. ovis 63/290	38	100	1000	1000	0.2	2	2

(This table was taken from ref. [54]).

### Multiplex PCR

To overcome the inherent disadvantage of cost of the test, multiplex PCR has been developed to detect viral, bacterial, and/or other infectious agents. The advantages of using multiplex PCR technique are that it minimizes expense and recognizes many pathogens at once [56]. These advances have resulted in the appearance of numerous publications regarding the application of multiplex PCR in the diagnosis of human brucellosis.

Lübeck PS *et al*. developed and applied a multiplex PCR assay for *Brucella* diagnostics based on the perosamine synthetase gene in 2003 [57]. El Kholy AA *et al*. also established a multiplex PCR technique using 2 sets of primers (B4/B5 and JPF/JPR) for the diagnosis of active human brucellosis in Egypt [58]. They found that this technique showed high sensitivity, specificity and accuracy, and could serve as important alternatives to culture methods for diagnosis of human brucellosis. Additionally, a multiplex PCR assay can be used to simultaneously detect and type *Brucella* species present in clinical samples. In 2007, Imaoka K *et al*. developed a multiplex PCR procedure to identify four major species of the genus *Brucella* in one reaction tube. Four pairs of primers targeting *bcsp31*, *omp2b*, *omp2a* and *omp31* genes were used. The specific amplification for each *Brucella* spp. examined in this study was achieved with these primers [59]. Other groups also reported robust and rapid multiplex PCR assays, which were able to identify and differentiate currently recognised *Brucella* species in a single test of less than an hour and a half [60]–[65]. The timely and accurate information provided by this assay would be valuable to trace sources of infection and may help in rapid diagnosis of human brucellosis. Furthermore, several multiplex PCRs have been described for identification of *Brucella* partly at the biovar level using different primer combinations. A 19-primer multiplex PCR specifically identified *B. neotomae*, *B. pinnipedialis*, *B. ceti*, and *B. microti* simultaneously. Also, this method was able to differentiate *B. abortus* biovars 1, 2, 4 from biovars 3, 5, 6, 9 [66]. A novel multiplex PCR assay for the rapid detection of *Brucella* genus at the species and at the biovar level has been described. The assay was shown to be ideal method for detection of *B. suis* at the biovar level and the differentiation of *B. suis*, *B. canis* and *B. microti*[67]. It is well knows that only a few biovars of *Brucella* species are pathogenic for humans, hence rapid identification of *Brucella* genus at the biovar level is necessary. Moreover, several multiplex PCRs have been described for the simultaneous detection of *Mycobacterium tuberculosis* (*M. tuberculosis*) complex and *Brucella* spp., targeting the *IS711*, *bcsp31* and *omp2a* genes for the identification of *Brucella* spp. and the *IS6110*, *senX3-regX3* and *cfp31* genes for the detection of the *M. tuberculosis* complex [68]–[70]. The results showed that this technique was a practical approach for the differential diagnosis between extrapulmonary tuberculosis and complicated brucellosis.

The primer pairs have substantial effect on the multiplex PCR efficiency. The presence of more than one primer pair in the multiplex PCR increases risk for primer-dimers. Thus, nonspecific products may be obtained [71]. Ideally, all the primer pairs in a multiplex PCR should not only eliminate non-specific PCR products, but also enable similar amplification efficiencies for their respective target. Therefore, the multiplex PCR requires laborious optimization [72].

### Nested and semi-nested PCR

The nested PCR means that two different pairs of PCR primers are used for a single locus. The first pair is an amplified sequence. The second pair of primers (nested primers) is complementary to the sequence amplified by the first pair primers and produces a second PCR product that will be shorter than the first one [73]. Same as nested, semi-nested PCR has two different pairs of PCR primers, but the second pair of primers has one primer identical to the first pair [74]. The nested PCR and semi-nested PCR amplify only the specific sequences sought and are more specific than the standard PCR. Recently, nested PCR and semi-nested PCR assays were developed for identifying *Brucella* in samples of human blood and then to explore their clinical practice for the diagnosis of human brucellosis.

Two nested PCR assays have been applied for the diagnosis of human brucellosis in Kuwait. Two pairs of primers derived from *IS711* were used. The results showed that the use of nested primers gave increased sensitivity and higher specificity providing a better molecular diagnostic approach for human brucellosis [75],[76]. Lin GZ *et al*. also reported a nested PCR for the laboratory diagnosis of human brucellosis [77]. Moreover, a semi-nested PCR for diagnosis of human brucellosis were developed and evaluated with whole blood. The primers were from *IS6501* and *bcsp31* genes [78]. We are now performing nested PCR combined with real-time PCR approach for the diagnosis of human brucellosis with the primers from *bcsp31* and *VirB11* genes. *B. abortus* and *B. melitensis* can be rapidly identified. The results have not been reported. According to the results, this assay was sensitive and could be used for the diagnosis of human brucellosis in the clinical laboratory.

However, the nested PCR and semi-nested PCR have some disadvantages. For example, the assays increased risk of primer dimerization and cross-react of PCR products. In addition, the nested PCR or semi-nested PCR will only identify a set of *Brucella* bacteria, not a single specific species.

### Other PCR-based assays

In addition to standard PCR and its derivatives (nested and semi-nested, multiplex and real-time PCR), there are other significant PCR-based assays have been developed in the last years. Fekete *et al*. developed the arbitrarily primed polymerase chain reaction (AP-PCR) to distinguish 25 different *Brucella* strains according to the banding patterns of their amplified DNA on agarose gels. The degrees of relatedness among these strains of the genus *Brucella* were revealed through the calculated similarity coefficients [79]. In 1994, AMOS PCR assays were used to identify vaccine strains from strains that cause infections based on the number and sizes of products amplified by PCR [80],[81]. In 1996, Tcherneva *et al*. reported the REP-PCR as a promising fingerprinting method for the evaluation of *Brucella* outbreak [82]. Also, various PCR-RFLPs display sufficient polymorphism to distinguish *Brucella* species and biovars, and can serve as tools for diagnostic, epidemiological, taxonomic, and evolutionary studies [83]. In addition, Multiple-locus variable-number tandem-repeat assays (MLVA) were used to study the molecular epidemiological characterization of *Brucella* isolates from humans [84]. PCR methods have been used successfully to identify all *Brucella* species and most of the biovars, offering an improvement over conventional molecular genotyping methods [85],[86]. Furthermore, Bruce-ladder multiplex PCR assay was evaluated using 625 *Brucella* strains. This method can differentiate in a single step all of the classical *Brucella* species, including strains from marine mammals and the S19, RB51, and Rev.1 vaccine strains [67]. Recently, the microfluidic Lab-on-Chip was also proposed as a rapid and specific detection method for the characterization of *Brucella* isolates [87],[88].

PCR-based assays were shown to be valuable tools for detecting *Brucella* strains. PCR approaches have several advantages for the diagnosis of human brucellosis, such as speed, safety, high sensitivity and specificity [89]. This technique might be considered complementary to the traditional methods and followed up by serology and/or culture [90]. However, its disadvantages such as the higher cost, issues of quality control and quality assurance must be further evaluated on clinical samples before PCR can be used in routine laboratory testing for human brucellosis [9],[91].

### Evaluation of PCR results for treatment and follow-up of patients

Most patients with brucellosis suffer a relapse after receive the duration and combination of antibiotic therapy. Therefore, it is necessary to evaluate the progress towards therapeutic failure or relapse [50]. The conventional methods are difficult for the diagnosis of these relapses. Several previous studies reported the applications of PCR for the diagnosis of post treatment follow-up and relapses. Queipo-Ortuño MI *et al*. examined the usefulness of PCR assay in post treatment follow-up and relapse of patients with brucellosis. They showed positive PCR tests for the relapse as well as negative once the relapse treatment was completed [26],[30]. Nimri LF obtained the positive PCR results in the relapse cases, indicating that the assay could be a useful tool to confirm a relapse in cases of a treated brucellosis [21]. Navarro E *et al.* also developed a real-time PCR assay to monitor the evolution of *Brucella melitensis* DNA load in blood during therapy and post-therapy follow-up in patients with brucellosis. This assay showed 100% analytical sensitivity for both initial infections and relapses [92]. Moreover, Mitka S *et al*. showed that PCR assays were negative in all follow-up samples from patients who had completed a successful treatment and were positive in all follow-up samples from patients who had relapses in the first year after therapy, including the times of the relapses [14]. However, dead phagocytosed bacteria may present in the circulating mononuclear cells in certain patients who have concluded successful treatment. Because PCR cannot differentiate between DNA from live and dead organisms, therefore, the ability to amplify the DNA of *Brucella* DNA from dead or phagocytized cells should be considered when interpreting the results.

## Conclusions

At present, PCR-based assays could allow rapid and more-sensitive identification of *Brucella* genus at the species and at the biovar level, compared with traditional techniques. The implementation of PCR-based assays into the clinical setting will likely improve therapeutic outcomes. However, PCR protocols lack standardization. As new methods for *Brucella* spp. identification and typing, PCR tests are still being developed and still await validation for use with clinical samples. For instance, the sensitivity and specificity of most PCR-based methods is associated with inhibitors in DNA samples such as EDTA, RNase or DNase, heme, heparin, phenol, and probably a host of other reagents. There is still a great deal of work required for standardization before any of these methods may be used in routine laboratory testing for brucellosis. Future studies should focus on the integration of these techniques into clinical decision making.

## Competing interests

The authors declare no conflicts of interest.

## Authors’ contributions

HY have made substantial contributions to conception, design and interpretation of data; YW, ZW, YZ, LB, YZ, CL and AM have been involved in drafting the manuscript or revising it critically for important intellectual content. All authors read and approved the final manuscript.
